# Sporadic Cryptosporidiosis Case-Control Study with Genotyping

**DOI:** 10.3201/eid1007.030582

**Published:** 2004-07

**Authors:** Paul R. Hunter, Sara Hughes, Sarah Woodhouse, Qutub Syed, Neville Q. Verlander, Rachel M. Chalmers, Kenton Morgan, Gordon Nichols, Nick Beeching, Keith Osborn

**Affiliations:** * University of East Anglia, Norwich United Kingdom;; † Communicable Disease Surveillance Centre – North West, Chester, United Kingdom;; ‡ Communicable Disease Surveillance Centre, London, United Kingdom;; § Singleton Hospital, Swansea, United Kingdom;; ¶ University of Liverpool, Liverpool, United Kingdom;; # Liverpool School of Tropical Medicine, Liverpool, United Kingdom;; ** United Utilities, Warrington, United Kingdom

**Keywords:** Cryptosporidiosis, *Cryptosporidium parvum*, *Cryptosporidium hominis*, case-control study, risk factors

## Abstract

We report a case-control study of sporadic cryptosporidiosis with genotyping of isolates from case-patients. A postal questionnaire was completed by 427 patients and 427 controls. We obtained genotyping data on isolates from 191 patients; 115 were *Cryptosporidium hominis*, and 76 were *C. parvum*. When all cryptosporidiosis cases were analyzed, three variables were strongly associated with illness: travel outside the United Kingdom, contact with another person with diarrhea, and touching cattle. Eating ice cream and eating raw vegetables were both strongly negatively associated with illness. Helping a child <5 years of age to use the toilet and the number of glasses of tap water drunk at home each day were also independently positively associated with risk. Eating tomatoes was negatively associated. For *C. hominis* infections, the strongly significant risk factors were travel abroad and changing diapers of children <5 years of age. For *C. parvum*, eating raw vegetables and eating tomatoes were strongly negatively associated with illness; touching farm animals was associated with illness.

## Abstract 2

Risk factors for Cryptosporidiosis in United Kingdom.

Cryptosporidiosis is due to infection by one or more species of the genus *Cryptosporidium*. Approximately 12 species are now recognized; two, *C. hominis* (previously known as *C. parvum*, genotype 1) and *C. parvum* (previously known as *C. parvum*, genotype 2), are the most important pathogens for humans (1). *C. hominis* is reported as being largely restricted to humans, and *C. parvum* is found in a wide range of animals (particularly cattle and sheep) as well as humans.

Most of what we know about the risk factors for *Cryptosporidium* infection was learned from outbreak investigations. Outbreaks have been associated with drinking water from public and private supplies, swimming in swimming pools, consumption of unpasteurized milk, and contact with farm animals, especially during farm visits. However, most cases of cryptosporidiosis are due to sporadic rather than outbreak-associated infections. Outbreaks represent <10% of all cases of *Cryptosporidium* infection (2,3), although a further proportion of cases will likely be associated with undetected outbreaks (4). Truly sporadic disease may not necessarily be due to the same causes (5). Only one substantive case-control study of sporadic cryptosporidiosis in the immune-competent population has been conducted in an industrialized nation (6). We are aware of only two other case-control studies of cryptosporidiosis in non–immune-compromised persons conducted in an industrialized nation (7,8); both studies were relatively small. One, from New Mexico, found an association with drinking water, although cases may possibly have represented an undetected outbreak (7). The other study, from Australia, found a borderline association with drinking bottled water. Concerns about the safety of the spring water on sale suggested that cases in this study may also have been part of an outbreak (8).

We report a large case-control study conducted in the North West Region of England and in Wales. The study was designed to investigate the etiology and epidemiology of sporadic cryptosporidiosis. The North West region has a history of a several large waterborne outbreaks of cryptosporidiosis over the past decade; Wales has not had any reported waterborne outbreaks.

## Methods

A case-control study was conducted in the North West of England and Wales from February 2001 to May 2002. The study received ethical approval from the Multi-centre Research Ethics Committee, relevant local Research Ethics Committees, and the Public Health Laboratory Service Research Ethics Committee. The principal hypotheses being tested in this study relate to the known epidemiology of outbreaks, namely, that sporadic cases of cryptosporidiosis are associated with drinking unboiled drinking water from public water supplies, swimming in a swimming pool, contact with animals, travel outside the United Kingdom, and contact with other persons with diarrheal illness. These hypotheses were constructed before the study began and were based on a review of previous U.K. outbreaks.

### Recruitment of Case-Patients and Controls

Participants were recruited through an enhanced program of surveillance of *Cryptosporidium* that had begun in the North West of England and Wales in December 2000. Details of confirmed cases identified by the enhanced surveillance were forwarded to Communicable Disease Surveillance Centre North West, through the consultant in Communicable Disease Control in each health authority.

Cases were defined as laboratory-confirmed *Cryptosporidium* in a resident of Wales or the North West region with diarrhea in the 2 weeks before a sample was taken, and which was not part of a formal outbreak investigation. All case-patients reported to Communicable Disease Surveillance Centre North West within 4 weeks of the date of report to the health authority were invited to take part in the study. Reports of patients in whom diarrhea had occurred more than 4 weeks previously were excluded because these patients may have had difficulty accurately recalling their activities before becoming ill.

A control was defined as a person who had not had diarrhea in the 2 weeks before completing a questionnaire. Controls were chosen to be within the same age band as the patients and within the same location; they were drawn from the same family physician’s practice or a neighboring practice. The age bands chosen were: <5 years of age, 5–16 years, and >16 years. Expecting control participation to be comparatively low, we attempted to recruit up to eight controls for each participating patient. We contacted the practice initially by mail. If no response was received, we contacted the practice manager by telephone. Each practice was approached only once in this way whether or not it had offered controls for our study and no matter how many cases had occurred in the practice. In many areas with high numbers of cases or low responses by practices, controls were not obtainable for many case-patients. Consequently, controls cannot be considered to be matched to cases. Rather, the control group was designed to be broadly comparable in age distribution to case-patients.

A total of 662 patients and 820 controls were invited to take part in the study. They received a questionnaire and an accompanying information leaflet by mail. If no response had been received after 2 weeks, a second questionnaire was sent. After this time, we assumed the person did not want to take part in the study.

Questionnaires were developed for both adult and child patients and controls. A person <16 years of age was defined as a child, and a person >16 years was defined as an adult. Finally, *Cryptosporidium* genotype data, held at the Cryptosporidium Reference Laboratory, was linked back to the recruited case-patients for epidemiologic analysis. Copies of the questionnaire can be obtained from the corresponding author.

### Genotyping

At the start of the study, all laboratories in the North West and in Wales were asked to send *Cryptosporidium*-positive stools to the Public Health Laboratory Service Cryptosporidium Reference Unit in Swansea for typing. To confirm the identification of *Cryptosporidium* at this unit, fecal smears were stained by using a modified Ziehl-Neelsen stain (9) and inspected by bright-field microscopy or by using an auramine phenol method (10) and inspected by fluorescence microscopy. Before DNA extraction, oocysts were purified from the feces by using salt flotation (11). The *Cryptosporidium* genotype was investigated by using polymerase chain reaction–restriction fragment length polymorphism to identify polymprophisms within the *Cryptosporidium* oocyst wall protein and *SSUrDNA* gene loci (12). These two methods are the routine methods for genotyping *Cryptosporidium* at the U.K. Reference Laboratory.

### Data Analysis

Data entry was performed by using EpiInfo (Version 6.04d, Centers for Disease Control and Prevention, Atlanta, GA). Initial analyses on the clinical severity and initial symptoms were conducted by using SPSS (SSPS Inc., Chicago, IL). Statistical modeling of risk factors was performed by the Public Health Laboratory Service Statistics Unit, using EpiInfo and GLIM (Generalised Linear Interactive Modelling [13]).

For etiologic analyses, each potential risk factor was considered singly by its odds ratio (OR) estimate (and 95% confidence interval [CI]). Continuity corrected chi-square tests or Fisher exact test was used when data were sparse. Dose-response rate was estimated by using chi-square tests for trends.

Variables that were positively or negatively associated with illness (p < 0.2) were included in a logistic regression model. However, all the variables could not be added, as too many existed for the statistical package to handle. Thus, all positive and some negative factors were included in the initial model. The least significant variable was removed and another negative factor included. This process continued until all the protective factors had been included. Then a sequence of models was fitted; on each occasion, the least significant variable was dropped.

Whether a child ate soil was the first variable removed because it had the most missing data for a nonsignificant variable and its removal resulted in many more observations being available for model estimation. Terms were assessed by comparing nested models by using likelihood ratio tests. Nonsignificant variables (p > 0.05) were removed one at a time from models, with the least significant ones being removed first. This process resulted in a final multivariable model, with most variables being significant or close to significant. The only variable that was retained whether or not it was significant was age.

Multivariable analyses were conducted separately on cases genotyped as either *C. hominis* and *C. parvum*. The set of variables for inclusion into initial multivariable models was determined by using all the data, as discussed above. Only cases with complete variables could be included in the final models.

## Results

Completed questionnaires were received from a total of 427 patients (65% response rate) and 427 controls (52% response rate). By chance, the number of patients and controls was equal. Of the controls, 27 (6%) had had diarrhea in the 2 weeks before completing the questionnaire and were excluded from the analysis. Of the patients, 191 (45%) had had strains sent to the Cryptosporidium Reference Laboratory, and the genotype was therefore known; 115 were *C. hominis* and 76 were *C. parvum*.

The median age for recruited case-patients and controls was 12 years. By sex, 48% of cases and 48% of controls were male. The age distribution of patients and controls is shown in Table 1, which gives the average age for 5- or 10-year age bands. The median age for controls and all patients was 12 years, although single variable analysis of age as a continuous variable indicated an association with illness (p = 0.007) with decreasing risk for illness with increasing age (estimated OR 0.991, 95% CI 0.985–0.998). The Table A1 shows the single variable analysis results for selected variables.

**Table 1:** Age distribution of controls and case-patients

Age group, y	Control n (%)	All cases n (%)	*C. hominis* n (%)	*C. parvum* n (%)
0–4	98 (24.6)	118 (27.7)	23 (20.0)	25 (32.9)
5–9	69 (17.3)	73 (17.1)	17 (14.8)	14 (18.4)
10–14	53 (13.3)	33 (7.7)	7 (6.1)	6 (7.9)
15–19	12 (3.0)	24 (5.6)	7 (6.1)	8 (10.5)
20–24	6 (1.5)	21 (4.9)	10 (8.7)	5 (6.6)
25–29	11 (2.8)	29 (6.8)	11 (9.6)	5 (6.6)
30–34	6 (1.5)	35 (8.2)	14 (12.2)	5 (6.6)
35–39	20 (5.0)	23 (5.4)	10 (8.7)	1 (1.3)
40–44	21 (5.3)	16 (3.8)	1 (0.9)	1 (1.3)
45–49	26 (6.5)	9 (2.1)	2 (1.7)	1 (1.3)
50–54	15 (3.8)	9 (2.1)	0	0
55–59	17 (4.3)	11 (2.6)	4 (3.5)	4 (5.3)
60–64	13 (3.3)	5 (1.2)	4 (3.5)	0 (0.0)
65–69	12 (3.0)	10 (2.3)	1 (0.9)	0 (0.0)
70–74	9 (2.3)	4 (0.9)	2 (1.7)	1 (1.3)
75–79	7 (1.8)	1 (0.2)	0	0
80–84	1 (0.3)	2 (0.5)	1 (0.9)	0
85–90	2 (0.5)	3 (0.7)	1 (0.9)	0

The age distributions of patients with infections from the two *Cryptosporidium* species differed markedly. The median age for persons with *C. hominis* infection was 21 years; for *C. parvum*, it was 9 years (p = 0.0036, Mann-Whitney U test) (Table 1). This finding was largely due to a second peak of *C. hominis* infections in persons in their 20s and 30s.

Regarding clinical details for patients, 251 (59%) reported fever, 410 (96%) abdominal pain, 279 (65%) vomiting, 49 (11%) bloody diarrhea, and 130 (30%) other symptoms. Sixty-one patients (14%) were admitted to hospital with a median 3-day stay (range 1–9). Persons infected with *C. hominis* or *C. parvum* had no significant differences in reported symptoms or hospital admissions.

The duration of illness for all patients (Figure, part A) showed a mean of 12.7 days and median of 11 days. For patients with *C. hominis*, the mean duration was 13.5 days (standard deviation [SD] 9.93, median 12.5) (Figure, part B). For *C. parvum*, mean duration was 11.33 days (SD 5.29, median 10.5) (Figure, part B). Levene’s Test for Equality of Variances showed that variance of duration for *C. parvum* was significantly lower than for *C. hominis* (F = 8.312, p = 0.005). However, the difference in median duration was not significant.

**Figure:**
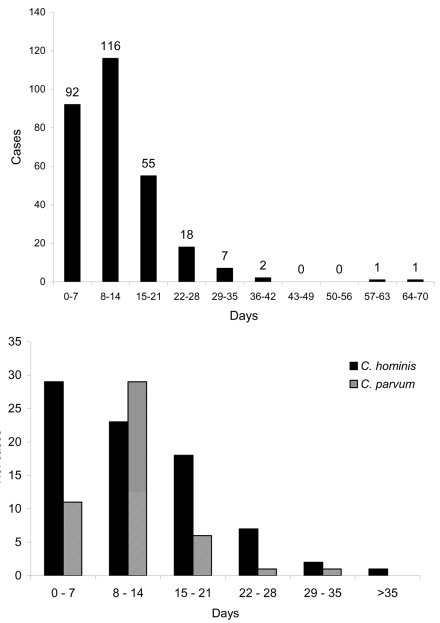
A, duration of illness, all patients. B, duration of illness for Cryptosporidium hominis and C. parvum patients.

Table 2 shows the multivariable results for all cases, estimated from 552 observations. In this model, the health authority, travel outside the United Kingdom (OR 5.650, p < 0.001), contact with another person with diarrhea (OR 4.614, p << 0.001), and touching any cattle (OR 3.876, p = 0.003) were highly significantly positively associated with risk. Toileting contact with a child <5 years of age (OR 1.851, p = 0.025) and the number of glasses of unboiled water drunk at home (OR 1.135 per glass, p = 0.019) were also positively associated. Eating ice cream (OR 0.472, p = 0.001), raw vegetables (OR 0.532, p = 0.004), and eating tomatoes (OR 0.616, p = 0.035) were negatively associated.

**Table 2:** Final multivariable model, all data^a,b^

Variables	Adjusted OR	95% CI	p value
Health authority			
A	1.000		0.004
B	0.125	0.041–0.382	
C	n.e.	n.e.	
D	0.482	0.166–1.398	
E	1.610	0.247–10.49	
F	0.225	0.080–0.635	
G	0.326	0.068–1.552	
H	0.921	0.261–3.250	
I	n.e.	n.e.	
J	0.310	0.117–0.822	
K	316.600	0–∞	
L	0.175	0.012–2.566	
M	0.377	0.130–1.097	
N	1.203	0.289–4.999	
O	0.367	0.11–1.145	
P	0.562	0.134–2.354	
Q	198.400	0–∞	
R	0.449	0.146–1.383	
S	0.206	0.053–0.804	
T	0.366	0.078–1.720	
U	0.546	0.207–1.443	
Age			
Travel outside UK			
Y	5.650	2.861–11.160	< 0.001
N	1.000		
Contact with another person with diarrhea			
Y	4.614	2.449–8.691	< 0.001
N	1.000		
Touch any cattle			
Y	3.876	1.4196–10.04	0.003
N	1.000		
Usually wash before eating raw fruit and vegetables			
Always	1.000		0.108
Usually	0.966	0.605–1.543	
Sometimes	0.746	0.436–1.274	
Never	2.478	0.965–6.362	
Toileting contact with child <5 y			
Y	1.851	1.079–3.175	0.025
N	1.000		
Number of glasses of unboiled water drunk at home			
Eat ice cream			
Y	0.472	0.299–0.746	0.001
N	1.000		
Eat raw vegetables			
Y	0.532	0.346–0.820	0.004
N	1.000		
Eat tomatoes			
Y	0.616	0.392–0.969	0.035
N	1.000		

^a^Estimated from 552 observations (261 case-patients and 291 controls). ^b^OR, odds ratio; CI, confidence intervals; n.e., not estimable.

Table 3 shows the final model for cases of *C. hominis*, estimated from 433 observations. Health authority of residence, travel outside the United Kingdom (OR 6.841, p < 0.001) and diaper-changing contact (OR 3.991, p < 0.001) were strongly associated with infection. Sitting or sleeping on the ground (OR 0.241, p = 0.027), the number of persons 5–15 years of age living with the person (OR 0.639 per person, p = 0.037), eating fresh fruit (OR 0.222, p = 0.027), and the likelihood of washing fresh fruit and vegetables before eating (p = 0.022) were negatively associated with risk.

**Table 3:** Final multivariable model for *Cryptosporidium hominis* infection^a,b^

Variables	Adjusted OR	95% CI	p value
Health authority			
A	1		< 0.001
B	0.030	0.003–0.335	
C	n.e.	n.e.	
D	0.781	0.206–2.960	
E	0.002	0–∞	
F	0.169	0.034–0.836	
G	0.277	0.022–3.516	
H	0.229	0.019–2.734	
I	n.e.	n.e.	
J	0.072	0.011–0.456	
K	n.e.	n.e.	
L	0.398	0.025–6.396	
M	2.116	0.573–7.809	
N	5.321	1.098–25.78	
O	0.169	0.017–1.685	
P	0.001	0–∞	
Q	n.e.	n.e.	
R	0.126	0.020–0.809	
S	0.408	0.065–2.539	
T	1.488	0.273–8.104	
U	1.015	0.288–3.579	
Age			
Travel outside UK			
Y	6.841	2.622–17.850	< 0.001
N	1.000		
Spend time sleeping or sitting outside on the ground			
Y	0.241	0.060–0.968	0.027
N	1.000		
Diaper changing contact with a child <5 y of age			
Y	3.991	1.848–8.618	< 0.001
N	1.000		
Usually wash before eating raw fruit and vegetables			
Always	1.000		0.022
Usually	0.347	0.159–0.757	
Sometimes	0.967	0.437–2.139	
Never	1.337	0.387–4.629	
No. of times swum in a toddler pool			
No. of persons 5–15 y of age living with you			
Eat fresh fruit			
Y	0.222	0.058–0.852	0.027
N	1.000		

^a^Estimated from 433 observations (82 case-patients and 351 controls). ^b^OR, odds ratio; CI, confidence interval; NE, not estimable.

The model in Table 4 shows the results for cases of *C. parvum*, estimated from 392 observations. Eating raw vegetables (OR 0.222, p = 0.001) and tomatoes (OR 0.317, p = 0.005) was negatively associated with illness; touching any farm animal (OR 2.653, p = 0.028) was associated with illness.

**Table 4:** Final multivariable model for *Cryptosporidium parvum* infection^a,b^

Variables	Adjusted OR	95% CI	p value
Health authority			
A	1		< 0.001
B	0.296	0.039–2.249	
C	n.e.	n.e.	
D	0.0001	0–∞	
E	0.0002	0–∞	
F	0.118	0.009–1.552	
G	0.0006	0–∞	
H	0.745	0.050–11.17	
I	n.e.	n.e.	
J	0.155	0.017–1.367	
K	0.00005	0–∞	
L	0.0002	0–∞	
M	0.981	0.136–7.082	
N	2.390	0.308–18.56	
O	0.0002	0–∞	
P	0.425	0.028–6.360	
Q	n.e.	n.e.	
R	1.239	0.186–8.260	
S	0.0001	0–∞	
T	0.643	0.043–9.545	
U	2.260	0.398–12.83	
Age			
Touch or handle farm animals			
Y	2.653	1.113–6.323	0.028
N	1.000		
Eat tomatoes			
Y	0.317	0.140–0.719	0.005
N	1.000		
Eat raw vegetables			
Y	0.222	0.086–0.572	0.001
N	1.000		
^a^Estimated from 392 observations (55 case-patients and 337 controls). ^b^OR, odds ratio; CI, confidence interval; n.e., not estimable.			

## Discussion

Our study is the first prospective epidemiologic study of sporadic cryptosporidiosis that has investigated independent risk factors for *C. hominis* and *C. parvum* infections. No significant differences were found between initial symptoms, severity of illness, or duration of hospital stay in persons infected with either *C. hominis* or *C. parvum* infections. No significant differences were found between median duration for *C. hominis* and *C. parvum*; however, the variation of duration for *C. hominis* was significantly higher than for *C. parvum i*nfections. This finding suggests that *C. hominis* infections may be less predictable in terms of duration and more prone to extremes than *C. parvum*.

The main risk factors identified—travel abroad, contact with a patient, and touching cattle—are broadly similar to those identified by Robertson and colleagues (6). Strongly significant negative factors were eating ice cream and eating raw vegetables. Factors significant at the 0.05 level were toileting contact with a child <5 years of age and number of glasses of unboiled water drunk at home. Eating tomatoes was negatively associated.

Health authority of residence was strongly significant in all models. However, given that we found that the ability to recruit controls differed between health authorities, much of this difference may be artifactual. Nevertheless, health authority of residence was retained in all models in the event that other risk factors varied in relation to locality of residence. The issue of geographic variation in cryptosporidiosis will be included in a subsequent report.

With regard to the main hypotheses under investigation, travel outside the United Kingdom, contact with other people with diarrhea, and contact with animals were all strongly associated with *Cryptosporidium* infection. Robertson et al. (6) also identified travel outside Australia as a risk factor. However, they suggested that OR may be inflated because of ascertainment bias of patients, which applies to our study as well. A general practitioner may be more likely to request a fecal sample from a patient with diarrhea who has traveled abroad. In addition, previous research indicates that most laboratories in the North West of England and Wales routinely screen for *Cryptosporidium* oocysts if the patient is known to have traveled outside of the United Kingdom (14). When analysis is restricted to cases where the species was known, travel outside the United Kingdom was significant for *C. hominis* infection but not for *C. parvum*. The relationship between *C. hominis* infection and overseas travel has been noted previously (15,16).

The risk for infection increased significantly upon contact with cattle when all patients were compared to controls, and for *C. parvum* alone but not for *C. hominis* alone. Previous research has associated farm animal contact with outbreaks of *Cryptosporidium*; moreover, calf contact and lamb contact have been identified as risk factors for sporadic infection (6). Several outbreaks have also been associated with farm visits within the United Kingdom. The risk for contact with other farm animals was not significant. The association with *C. parvum* but not *C. hominis* is also consistent with previous findings (15,16). Our study was conducted during an epidemic of foot and mouth disease, when access to the countryside and contact with farm animals were severely restricted for a large period (17), a fact that makes the cattle association even more dramatic. No significant association was found between ownership of or contact with domestic pets and sporadic infection. Although some researchers have suggested pets may present a risk (18), other studies indicate that pets are not a major risk factor for acquiring *Cryptosporidium* (19). Indeed, previous research has found various types of domestic animal contact to be protective (6).

One variable, number of glasses of unboiled water drunk at home, was significant in the model with all patients. This water consumption variable was the only one to be included in one of the multivariable models. The Australian study also found no association with drinking publicly supplied water (6). However, one of the two water catchment areas in this study was highly protected, with no livestock farming. The nature of the water catchment areas in Australia might preclude generalizing its results to other parts of the world. Interpreting this finding is difficult. Few of the drinking water variables associated with risk from water consumption were significant in the single variable analysis (Table A1). Neither drinking unboiled tap water nor use of a water filter was significant, which suggests that drinking water from public supplies was not an important risk factor (20). In the single variable analysis, number of glasses of bottled water drunk was also associated with risk for infection, although whether or not persons drank bottled water was not associated with risk. We suggest that the significant association with amount of unboiled water drunk may be an artifact attributable to recall bias either because the patient believes that his or her illness was waterborne (21) or because the person has been drinking more water as a result of illness. Our findings suggest that drinking tap water does not appear to be of major importance for sporadic disease.

The remaining risk factor included in the major hypotheses we tested, use of swimming pools, did not achieve significance, although number of times one swam in a toddler pool almost reached significance in the model for *C. hominis*. Use of a toddler pool and number of times swum in a swimming pool, but not use of a swimming pool, were significant in the single variable analyses (Table A1). Swimming pool use has previously been associated with many outbreaks of *Cryptosporidium* in the United Kingdom and elsewhere, and use of a toddler pool has been associated with sporadic cases (6). The importance of swimming pool exposure as a risk factor for sporadic cryptosporidiosis was suggested by Hunter and Quigley (22). They demonstrated a protective effect of swimming pool use in an outbreak associated with drinking water and suggested that this finding was due to immunity from an increased risk for sporadic disease in persons who go swimming.

In addition to the main hypotheses, a number of other associations were detected. These included a negative association with eating raw vegetables and tomatoes in the model with all patients and *C. parvum* only, a negative association with eating fresh fruit for *C. hominis*, a negative association with eating ice cream in the model with all patients, and an association with toileting children <5 years of age in the all-case model and diaper-changing contact in the *C. hominis* model. Also in the *C. hominis* model, spending time sleeping or sitting outside on the ground was associated with infection, the number of persons 5–15 years of age living in the same home was negatively associated with infection, and usually washing raw fruit and vegetables before eating had a protective effect.

The negative association with eating raw vegetables is also consistent with previous studies, which have suggested a protective effect from eating raw vegetables (6,18). Whether this represents the effect of immunity through repeat exposure by this route or through another mechanism is unclear (22,23).If the immunity hypothesis is correct, the fact that eating raw vegetables was strongly negatively associated with *C. parvum*, but not *C. hominis*, infection would suggest contamination of raw vegetables with animal-derived fecal material.

The negative association with ice cream was unexpected. In the single variable analysis, consuming other dairy products such as uncooked soft cheese, uncooked hard cheese, and cream were also negatively associated with illness. Unpasteurized milk products have previously been associated with *Cryptosporidium* infections, and consuming such products was identified as a risk factor for sporadic cases of infection in Adelaide (6). However, in the United Kingdom, unpasteurized milk is not used in ice-cream production, so this association is difficult to explain. We investigated the possibility that this finding was due to the different times of the year that patients and controls were recruited. However, in all but 1 month, controls were more likely to report ice cream consumption than patients were. A recently published case-control study on risk factors for giardiasis in the South West of England also reported a negative association with ice cream consumption (24).

Associations of toileting contact with children <5 years (all patients) and diaper-changing contact (*C. hominis*) were independent of whether the children were being helped to use the toilet or having their diapers changed had diarrhea. This observation would suggest that asymptomatic carriage of *C. hominis* may be common in very young children even in the absence of symptoms. Asymptomatic carriage of *C. hominis* may be the main reservoir of infection.

In conclusion, we showed that the main risk factors for *C. parvum* infection (contact with cattle) and *C. hominis* (travel abroad and changing diapers) differ. We also showed that when the case group includes both *C. parvum* and *C. hominis* as well as cases in which the species is not known, the risk factors vary again (travel abroad and contact with a case-patient). Although restricting analysis to cases where species is known reduces the power of the study by having fewer cases, analyses conducted on populations of patients that contain two pathogens with different epidemiologic features may mask species specific risk factors. Future studies of the epidemiology of and risk factors for cryptosporidiosis should ensure that strains are speciated adequately.

## Appendix

**Table A1:** Single variable analysis (chi-square test or Fisher exact test) of significant and selected nonsignificant variables^a^

Variables	Case-patients	Controls	OR	95% CIs	p value
General information					
Sex					
M	204	190	1.02	0.76–1.35	0.967
F	222	210			
Have a medical condition known to affect immunity					
Y	17	3	5.67	1.54–4.78	0.004
N	376	376			
No. of persons 5–15 years living with you					
0	115	63			< 0.001^b^
1	115	103	0.61	0.40–0.94	
2	39	53	0.40	0.23–0.70	
3	12	11	0.60	0.23–1.59	
4	2	4	0.27	0.00–21.70	
>5	0	1	0.00	0.00–21.70	
Travel outside UK					
Y	99	24	4.74	2.89–7.85	< 0.001
N	327	376			
Gardening other than watering					
Y	62	90	0.61	0.42–0.89	0.009
N	345	306			
Swimming pool use					
Swim in a swimming pool					
Y	172	147	1.15	0.86–1.55	0.362
N	252	248			
No. of times swum in a swimming pool					
0	252	248			< 0.001^b^
1	38	46	0.81	0.50–1.33	
2	39	52	0.74	0.46–1.19	
3	18	17	1.04	0.50–2.18	
4	13	11	1.16	0.48–2.84	
>5	53	18	2.90	1.60–5.29	
Use a toddler pool					
Y	76	50	1.52	1.01–2.28	0.043
N	345	344			
No. of times swum in toddler pool					
0	345	344			0.001^b^
1	16	16	1.00	0.47–2.14	
2	21	22	0.95	0.49–1.84	
3	5	4	1.25	0.27–6.33	
4	6	3	1.99	0.42–12.41	
>5	18	1	17.95	2.80–749.91	
Contact with animals					
Touch or handle any farm animals					
Y	70	43	1.65	1.07–2.54	0.021
N	344	348			
Touch any cattle					
Y	22	8	2.69	1.11–6.70	0.024
N	392	383			
Domestic pets living in home					
Y	207	214	0.82	0.62–1.09	0.180
N	219	186			
Contact with case-patients					
Anyone else in previous 2 weeks in house ill with diarrhea					
Y	83	28	3.20	1.98–5.20	< 0.001
N	334	361			
Contact with another person ill with diarrhea					
Y	91	24	4.27	2.59–7.10	< 0.001
N	324	365			
Contact with children <5 y					
Toileting contact with a child <5 y					
Y	86	52	1.69	1.14–2.51	0.008
N	341	348			
Diaper-changing contact with a child <5 y					
Y	71	48	1.46	0.96–2.22	0.073
N	356	352			
Drinking water consumption					
Did you drink unboiled tap water at home					
Y	347	342	0.82	0.54–1.23	0.359
N	67	54			
No. of glasses of tap water drunk a day					
0	67	54			0.037^b^
1	54	65	0.67	0.39–1.15	
2	57	74	0.62	0.37–1.05	
3	65	88	0.60	0.36–0.99	
4	47	57	0.66	0.38–1.16	
>5	73	33	1.78	1.00–3.19	
Use a water filter at home					
Y	39	34	1.11	0.66–1.85	0.771
N	373	360			
Disruption to your water supply at home					
Y	27	25	1.19	0.65–2.18	0.650
N	321	353			
Any water discoloration					
Y	22	16	1.33	0.6–6–2.72	0.490
N	392	380			
Any altered taste to the water					
Y	14	4	3.43	1.03–12.57	0.040
N	400	392			
Any loss of water pressure					
Y	11	13	0.80	0.33–1.95	0.751
N	403	383			
Any other problems with water					
Y	17	11	1.50	0.65–3.49	0.400
N	397	385			
Did you drink unboiled tap water at somewhere other than home					
Y	212	193	1.16	0.85–1.58	0.352
N	153	162			
Did you use ice cubes					
Y	120	113	1.05	0.76–1.45	0.813
N	277	274			
No. of times ice cubes were used					
0	277	274			0.944^b^
1	13	12	1.07	0.45–2.56	
2	17	24	0.70	0.35–1.39	
3	15	20	0.74	0.35–1.55	
4	19	9	2.09	0.88–5.08	
>5	30	31	0.96	0.55–1.68	
Drink any bottled water					
Y	164	148	1.13	0.83–1.52	0.457
N	228	232			
No. of glasses of bottled water a day					
0	228	232			0.018^a^
1	31	49	0.64	0.38–1.07	
2	34	37	0.94	0.55–1.59	
3	12	12	1.02	0.42–2.48	
4	16	9	1.81	0.74–4.53	
>5	21	8	2.67	1.10–6.71	
Consumption of raw vegetables or salads					
Eat lettuce					
Y	161	186	0.75	0.55–1.01	0.057
N	212	183			
Eat other green salad					
Y	165	204	0.61	0.45–0.83	0.001
N	194	146			
Eat tomatoes					
Y	195	249	0.54	0.39–0.73	< 0.001
N	184	126			
Eat coleslaw					
Y	83	116	0.62	0.44–0.89	0.007
N	267	233			
Eat raw vegetables					
Y	94	157	0.45	0.33–0.63	< 0.001
N	259	196			
Eat fresh fruit					
Y	332	361	0.36	0.20–0.63	< 0.001
N	54	21			
Consumption of dairy products					
Eat uncooked soft cheese					
Y	85	116	0.65	0.46–0.91	0.012
N	270	238			
Eat uncooked hard cheese					
Y	243	281	0.59	0.42–0.83	0.002
N	125	85			
Eat ice cream					
Y	249	284	0.55	0.39–0.78	< 0.001
N	127	80			
Eat or drink cream					
Y	86	124	0.60	0.42–0.84	0.003
N	259	223			
Area of residence					
Health authority					
A	40	19			< 0.001
B	14	46	0.14	0.06–0.32	
C	0	0	n.e.	n.e.	
D	40	37	0.51	0.25–1.04	
E	8	4	0.95	0.25–3.55	
F	32	58	0.26	0.13–0.53	
G	7	7	0.48	0.15–5.55	
H	17	10	0.81	0.31–2.09	
I	0	0	n.e.	n.e.	
J	42	51	0.39	0.20–0.77	
K	1	1	0.48	0.83–8.01	
L	5	4	0.59	0.14–2.47	
M	43	29	0.70	0.34–1.45	
N	24	10	1.14	0.46–2.85	
O	27	23	0.56	0.26–1.22	
P	15	6	1.19	0.40–3.54	
Q	1	0	n.e.	0.0004–¥	
R	24	23	0.50	0.22–1.09	
S	8	17	0.22	0.08–0.61	
T	5	10	0.24	0.07–0.79	
U	74	45	0.78	0.40–1.51	

^a^OR, odds ratio; CI, confidence interval; n.e., not estimable. ^b^Chi-square test for trend.
